# Association between exposure to digital alcohol marketing and alcohol use: a systematic review and meta-analysis

**DOI:** 10.1016/S2468-2667(25)00219-1

**Published:** 2025-11

**Authors:** Scott I Donaldson, Alex M Russell, Kathryn La Capria, Amanda DeJesus, Emily Wang, Jamil Fayad, Jon-Patrick Allem

**Affiliations:** Institute for Nicotine and Tobacco Studies, Rutgers Biomedical and Health Sciences, New Brunswick, NJ, USA; Division of General Internal Medicine, Rutgers Robert Wood Johnson Medical School, New Brunswick, NJ, USA; Recovery Research Institute, Massachusetts General Hospital and Harvard Medical School, Boston, MA, USA; Institute for Nicotine and Tobacco Studies, Rutgers Biomedical and Health Sciences, New Brunswick, NJ, USA; Institute for Nicotine and Tobacco Studies, Rutgers Biomedical and Health Sciences, New Brunswick, NJ, USA; Institute for Nicotine and Tobacco Studies, Rutgers Biomedical and Health Sciences, New Brunswick, NJ, USA; Institute for Nicotine and Tobacco Studies, Rutgers Biomedical and Health Sciences, New Brunswick, NJ, USA; Institute for Nicotine and Tobacco Studies, Rutgers Biomedical and Health Sciences, New Brunswick, NJ, USA; Division of General Internal Medicine, Rutgers Robert Wood Johnson Medical School, New Brunswick, NJ, USA; Department of Health Behavior, Society and Policy, Rutgers School of Public Health, The State University of New Jersey, Piscataway, NJ, USA

## Abstract

**Background:**

Exposure to digital alcohol marketing content might be associated with pro-alcohol-related attitudes and behaviours, including the likelihood of initiating or sustaining the use of alcohol, especially among adolescents (aged <18 years) and young adults (aged 18–25 years). This study aimed to examine the relationship between exposure to digital alcohol marketing content and alcohol use outcomes.

**Methods:**

Alcohol-related, digital media-related, and marketing-related search terms were entered into six online databases: PubMed, Web of Science, Scopus, PsycINFO, Embase, and Communication & Mass Media. Peer-reviewed articles written in English, published between Jan 1, 2004, and Feb 1, 2025, were included in the search. Studies that were included measured self-reported exposure to digital alcohol marketing content; used an unexposed control group; measured past 30-day alcohol use, binge drinking, or susceptibility to use alcohol among never users; and provided raw data to compute odds ratios (ORs) or reported ORs in the manuscript. When available, adjusted odds ratios were included; otherwise, unadjusted estimates were computed from raw data. A multilevel random-effects meta-analysis was used to estimate ORs and 95% CI, and heterogeneity (*I*^2^) was calculated for each alcohol use outcome. Study quality and publication bias were assessed. The study protocol was registered on the Open Science Framework.

**Findings:**

The search identified 9913 articles. 96 articles were eligible for full-text review, of which 65 articles were removed based on the exclusion criteria. 31 studies were included in the final meta-analysis. The total sample size was 62 703 participants (32 314 [51·5%] female; 30 389 [48·5%] male, including 52 475 (83·7%) adolescents (aged 11–17 years) and 10 228 (16·3%) adults (aged ≥18 years). Participants exposed to digital alcohol marketing content, compared with those not exposed, had greater odds of reporting past 30-day alcohol use (19 studies, 46 361 participants; OR 1·75 [95% CI 1·39–2·20]; *I*^2^=91·0%), binge drinking (13 studies, 25 603 participants; 1·80 [1·22–2·67]; *I*^2^=95%), and susceptibility to use alcohol among never users (seven studies, 18 698 participants; 1·78 [1·29–2·46]; *I*^2^=88%).

**Interpretation:**

Findings demonstrated an association between exposure to digital alcohol marketing content and alcohol-related behaviours. Future research is needed to clarify the temporal order between exposure to digital alcohol marketing content and alcohol-related behaviours.

**Funding:**

The National Institute on Alcohol Abuse and Alcoholism of the National Institutes of Health.

## Introduction

Adolescent (aged 11–17 years) and young adult (aged 18–25 years) alcohol consumption is a serious public health concern associated with numerous consequences, such as physical harm, cognitive impairment, and increased risk for alcohol use disorder.^1^ Alcohol consumption represents a leading cause of preventable death and disability.^1^ In 2024, almost half (47·5%; 16·6 million) of young adults aged 18–25 years living in the USA consumed alcohol during the past 30 days, including 26·7% (9·3 million) that binged (ie, consumed five or more drinks for men or four or more drinks for women on a single occasion) over the same period.^2^ Of the 4·2 million Americans who consumed their first drink of alcohol in 2024, 40·5% (approximately 1·7 million) were between the ages of 12 years and 17 years.^2^ Alcohol use trajectories often escalate during adolescence and young adulthood when individuals are particularly susceptible to social and commercial influences, including marketing influences.^3^

The alcohol industry uses marketing to influence both current and future alcohol consumption.^4^ In particular, adolescents and young adults have been disproportionately affected by alcohol marketing, with studies linking marketing exposure to increased brand awareness, appeal of alcoholic products, positive use expectancies, and levels of consumption.^5–7^ For instance, a review of prospective cohort studies found that exposure to alcohol marketing was associated with subsequent drinking behaviours in adolescents and young adults.^6^ Additionally, evidence from experimental and cross-sectional studies has demonstrated positive associations between exposure to alcohol marketing and alcohol use behaviours among young adults.^8,9^

With the growing popularity of digital media among adolescents and young adults, marketing efforts have increased on digital platforms.^10^ For example, adolescents and young adults have reported greater exposure to digital alcohol marketing compared with adults.^9–12^ Social media platforms use algorithms, often driven by artificial intelligence, to personalise content and increase exposure to specific content (eg, pro-alcohol-related content) among users whose behaviours or previous engagement predict interest in such content.^13^ Additionally, digital alcohol marketing is poorly regulated, with many countries relying primarily on industry self-regulation with no independent oversight,^14^ clear enforcement mechanisms, or meaningful penalties for violations.^15,16^ Indeed, recent calls from WHO, and its member states, have underscored the urgency of addressing these regulatory gaps, emphasising the need for stronger national and international policies to restrict the digital marketing of health-harming products, including alcohol.^17^

Exposure to digital alcohol marketing content, defined in the current study as social media, web-based, or app-based content designed to increase alcohol use through promotional campaigns, branded content, or sponsorships, has been associated with increased alcohol consumption.^18^ However, research to date has not quantitatively summarised the pooled effect of exposure to digital alcohol marketing content across studies with diverse populations or explored how methodological features or study characteristics, including digital platform type, are associated with alcohol use behaviours. To gain a clearer understanding of the relationship between exposure to digital alcohol marketing content and alcohol use, we did a meta-analysis to estimate the overall association between exposure to digital alcohol marketing content, past 30-day alcohol use, binge drinking, susceptibility to use alcohol among never users, and lifetime alcohol use.

## Methods

### Search strategy and selection criteria

For this systematic review and meta-analysis, we followed the PRISMA guidelines (appendix pp 2–4).^19^ Past reviews were used to develop a list of digital media-related, alcohol-related, and marketing-related search terms.^18,20^ Next, a Boolean search string was entered into six electronic databases: PubMed, PsycINFO, Web of Science, Scopus, Embase, and Communication & Mass Media (appendix p 5). Peer-reviewed articles written in English, including dissertations, published between Jan 1, 2004, and Feb 1, 2025, were included in the search. Facebook, one of the oldest social media platforms, was launched in 2004, suggesting this period would be inclusive of the most relevant studies.

The definition of digital alcohol marketing content (ie, social media, web-based, or app-based content designed to increase alcohol use through advertising, promotions, and sponsorships) used in this study was informed by previous research.^18^ To be included in this analysis, a study needed to have the following: measured self-reported exposure (ie, any self-reported exposure frequency greater than “never”) to, or experimentally manipulated, digital alcohol marketing content; used an unexposed control group (ie, participants who were not exposed to, or reported not being exposed to, digital alcohol marketing content in their lifetime); measured past 30-day alcohol use, binge drinking (ie, lifetime or past 30 days), susceptibility to use alcohol among never users, or lifetime alcohol use; and provided raw data (eg, frequencies) to compute odds ratios (ORs) or reported ORs in the manuscript. A study was excluded from this analysis if it had only measured exposure to alcohol marketing content from offline media sources, including a billboard, retail store, poster, or magazine, used a control group that reported some degree of exposure to digital alcohol marketing content (ie, were exposed to digital alcohol marketing content at least once in their lifetime), measured non-alcohol use outcomes (eg, subjective norms), were opinion pieces or editorials, or measured exposure to anti-alcohol content (eg, health campaigns). If relevant statistics were not provided in the article, the corresponding author of the article was contacted. Contact with the corresponding author was attempted up to three times. If the corresponding author was non-responsive and a different last (ie, senior) author was listed, the last author was contacted up to three times, and the studies were excluded if the author was not contactable.

Covidence software was used for title and abstract screening and full-text review.^21^ Two reviewers (KLC and AD) independently coded articles in two phases. In the first phase, the titles and abstracts of the papers were assessed for relevance (*%*_agreement_=96·9%), including the presence of alcohol-related, digital media, and marketing-related search terms. In the second phase, the full articles were assessed on the inclusion and exclusion criteria (*%*_agreement_=88·9%). All articles were double-coded, and disagreements were discussed until a consensus was reached. The study protocol was registered on the Open Science Framework and is available online.

### Data analysis

Data extracted from the included articles were added to a table in Excel. Extracted data consisted of study design, location, population, age, gender, digital platform (ie, other web-based *vs* social media), exposure engagement (ie, passive *vs* active), exposure timeframe (ie, lifetime *vs* past 30 days), adjustment status (ie, whether estimates were adjusted for potential confounders), and alcohol use outcomes. Study design was coded as cross-sectional, longitudinal, or experimental. Location was coded at the country level. Sample size was coded to reflect the number of participants from each study. Population was coded to reflect adolescents (ie, age <18 years), young adults (ie, age 18–25 years), or adults (ie, age >25 years). Sex was coded as the percentage of female participants. Race and ethnicity were not extracted due to inconsistent or absent reporting across studies. Digital platform was coded as social media (eg, Facebook, Twitter, YouTube, Pinterest, GooglePlus, Tumblr, Instagram, or Snapchat) or web-based (eg, website, email, or phone-based applications). Engagement was coded as active engagement (ie, searching, posting, commenting, or liking digital alcohol-related marketing content) or passive engagement (ie, only viewing digital alcohol-related marketing). Exposure to digital alcohol marketing content was coded as lifetime or past 30 days. Exposure recall method was coded as open-ended, check box, yes or no, or scale. Alcohol use outcomes were coded as lifetime use, past 30-day use, binge drinking (ie, coded as >5 drinks as defined by the National Institute on Alcohol Abuse and Alcoholism),^22^ and susceptibility to use alcohol (eg, drinking intentions in the next year) among never users. This study did not account for sex differences (ie, >4 drinks for females is considered binge drinking) while coding the binge drinking outcome. For simplicity, a conservative estimate (ie, >5 drinks) was used regardless of sex to code for the presence of binge drinking.

To assess the association between design quality, sample breadth, and effect size, a rubric from previous research was used (appendix p 6).^23^ The rubric defined design quality on a continuum: cross-sectional and convenience, cross-sectional and representative, longitudinal and convenience, longitudinal and representative, experimental and convenience, and experimental and representative. Experimental designs that demonstrated potential causality between exposure to digital alcohol marketing content and alcohol use behaviours were ranked the highest. Sampling breadth was coded at the local, state, and national levels.

The Risk of Bias In Non-randomized Studies of Exposures (ROBINS-E) tool was used to assess potential sources of bias across seven domains.^24^ For this study, the following six domains were applied (see appendix pp 7–8 for the ROBINS-E risk of bias codebook): bias due to confounding, selection of participants, classification of exposures, missing data, measurement of outcomes, and selection of reported results. The domain related to deviations from intended exposures was excluded because most studies had designs that were cross-sectional. The assessments of risk of bias for each study (low risk, some concerns, or high risk) were done separately by two reviewers (SID and AMR) in duplicate, and then discussed to resolve discrepancy. Domain-level and overall judgements were informed by established guidance,^24^ with overall ratings reflecting the highest level of bias identified in any domain.

A random-effects meta-analysis was used to test the hypothesis that participants exposed to digital alcohol marketing content, compared with those not exposed, will have greater odds of reporting alcohol use behaviors.^25^ ORs were collected from each primary study or computed from raw data (eg, 2 × 2 contingency tables, frequencies, or sample sizes) using the practical meta-analysis calculator.^26^ Publications from the same dataset were treated as part of the same study cluster, meaning that their effect sizes were nested under a single study-level identifier. In cases with multiple exposure–outcome pairs (eg, different platforms), the most inclusive, mutually exclusive comparisons were selected to avoid duplication and overlapping samples. When exposure frequency was reported in more than two categories, exposure and outcome variables were dichotomised consistently across studies (eg, never *vs* monthly or weekly) to standardise effect sizes. Therefore, several studies reported multiple effect sizes, creating a statistical dependency known as the unit-of-analysis error.^27^ To account for this dependency, a multilevel generic inverse variance random-effects meta-analysis was used.^28^ A Knapp–Hartung (Tipton-style) small-sample correction was used in all models to ensure robust standard errors and appropriate degrees of freedom given the small number of studies.^29^

The heterogeneity of effect sizes (ie, the extent to which effect sizes vary between studies) was assessed using a restricted maximum-likelihood estimator for *t*^2^ and *I*^2^. Subgroup analyses, including demographic and methodological variables, were examined to assess their association with alcohol use outcomes. Subgroup variables might help explain why effect sizes differ from study to study, including differences in demographic characteristics and study design. A test for subgroup differences was performed using Q, df, and p values to detect statistical significance among categories within subgroup variables.

Publication bias (ie, the threat that excluding non-significant findings might impact the effect size estimates) was assessed in two ways. First, a Rucker’s limit meta-analysis was used to measure bias due to small-study effects.^30–32^ Second, a p-curve analysis was used to assess p-hacking (ie, when researchers modify a statistical analysis until non-significant results become significant).^33^ A p-curve analysis shows the number of effect sizes that were significant at p=0·05, p=0·04, p=0·03, etc. A true underlying effect is present when highly significant findings (p=0·01) are more likely than marginally significant findings (p=0·049).^34^ Additionally, sensitivity analyses were conducted for each outcome variable to compare effect size estimates from adjusted versus unadjusted models.

All meta-analysis procedures were performed in R, version 4.3.2, using the packages meta, metafor, and robvis.^35–37^

### Role of the funding source

The funders of the study had no role in study design, data collection, data analysis, data interpretation, or writing of the report.

## Results

**Figure 1:**
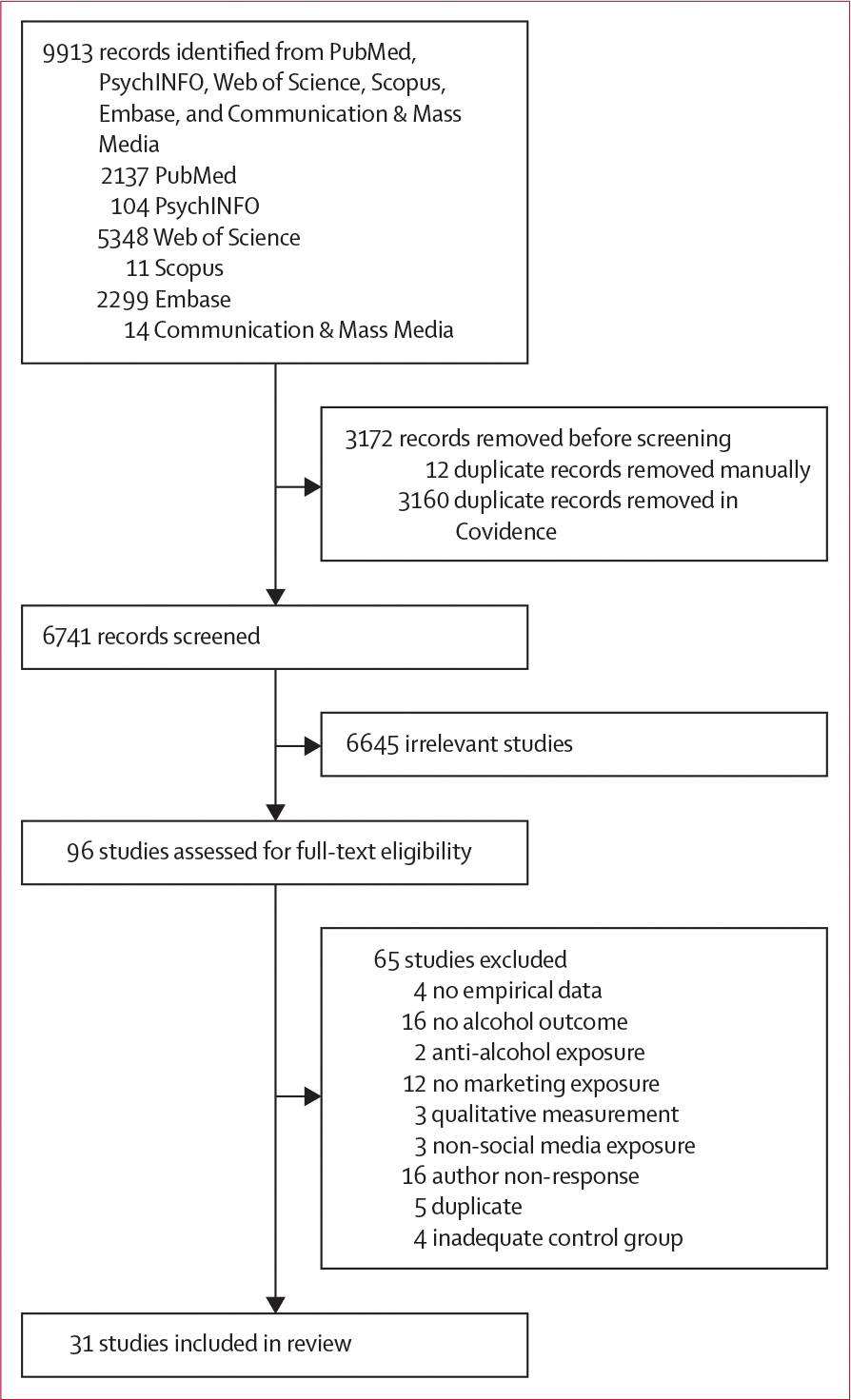
Study selection

A total of 9913 articles were identified using the keyword search strategy. After the removal of duplicate articles, 6741 articles were selected for title and abstract screening (figure 1). 96 articles were eligible for full-text review from which 65 articles were removed based on the exclusion criteria. The analytic sample for this meta-analysis comprised 31 articles.^38–68^

The total sample size was 62,703 participants (32,314 [51·5%] female; 30,389 [48·5%] male), including 52 475 (83·7%) adolescents (aged 11 to <18 years) and 10 228 (16·3%) adults (aged ≥18 years; table). 26 (83·9%) of 31 studies used cross-sectional research designs, four studies (12·9%) used longitudinal research designs, and one study (3·2%) used an experimental research design. Participants were represented across 17 countries, which were Australia, the USA, China, New Zealand, South Africa, the UK, Germany, Italy, India, Ireland, Japan, Mongolia, the Netherlands, Poland, Taiwan, Thailand, and Uganda.

**Figure 2:**
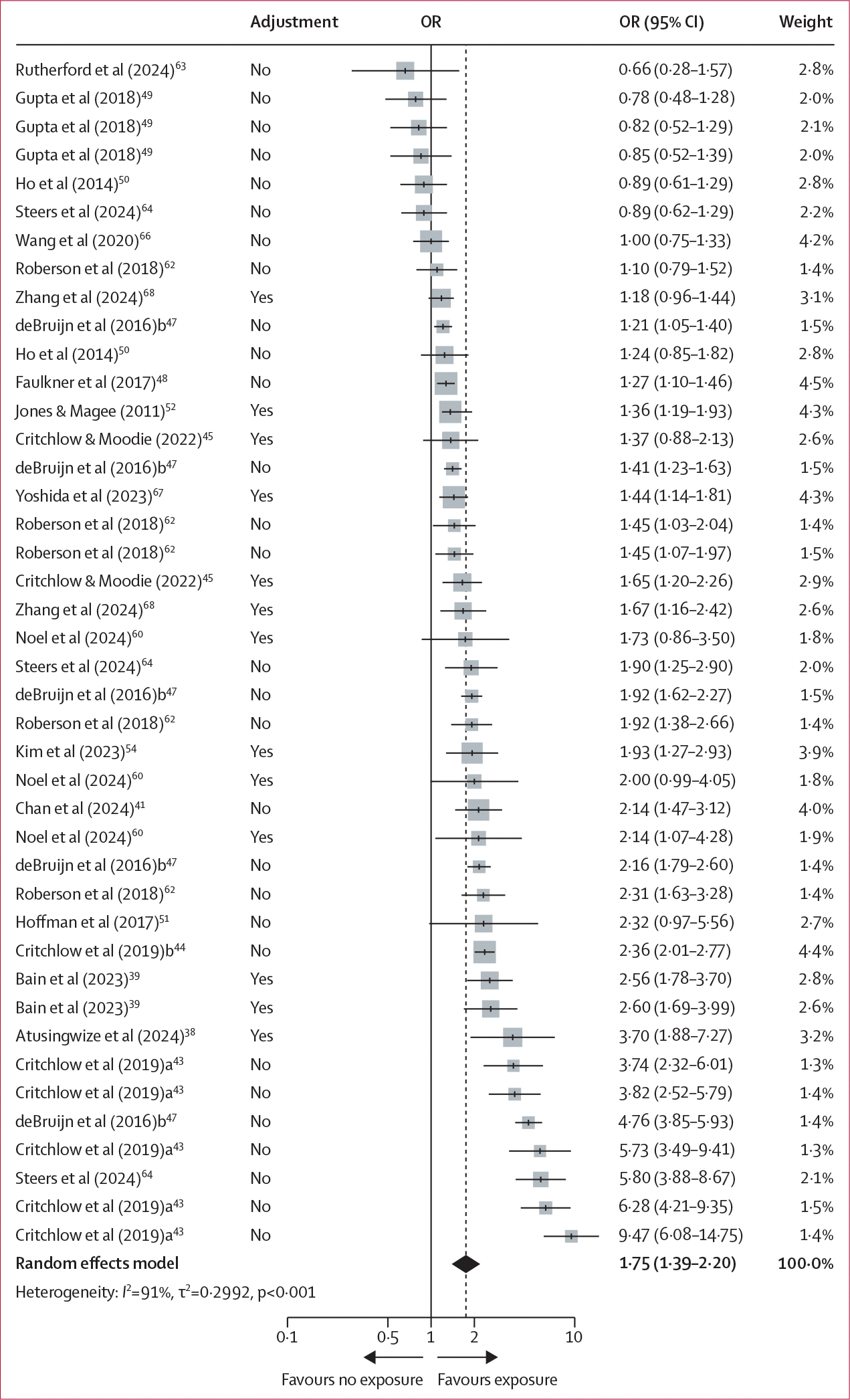
Forest plot of multilevel meta-analysis for exposure to digital alcohol marketing content and past 30-day alcohol use Adjustment=whether or not a covariate adjustment was used to compute the OR. OR=odds ratio.

Participants who were exposed to digital alcohol marketing content, compared with those who were not exposed, had greater odds of reporting past 30-day alcohol use (19 studies, 46 361 participants; OR 1·75 [95% CI 1·39–2·20]; *I*^2^=91·0%; figure 2). The estimated variance components were τ^2^_Level 3_=0·1560 and τ^2^_Level 2_=0·1432, corresponding to *I*^2^_Level 3_=48·6% of the total variation attributed to between-cluster, and *I*^2^_Level 2_=44·6% to within-cluster heterogeneity. The three-level model provided a significantly better fit compared with a two-level model with level 3 heterogeneity constrained to zero (χ_1_^2^=11·50; p<0·01). Subgroup analyses showed that participants exposed to digital alcohol marketing content in the past 30 days, compared with those who were exposed in their lifetime, had greater odds of reporting past 30-day alcohol use (appendix p 10). Similar associations with past 30-day alcohol use were found for participants exposed to digital alcohol marketing content on social media platforms compared with other web-based platforms, and adolescents compared with adults (appendix p 10).

**Figure 3:**
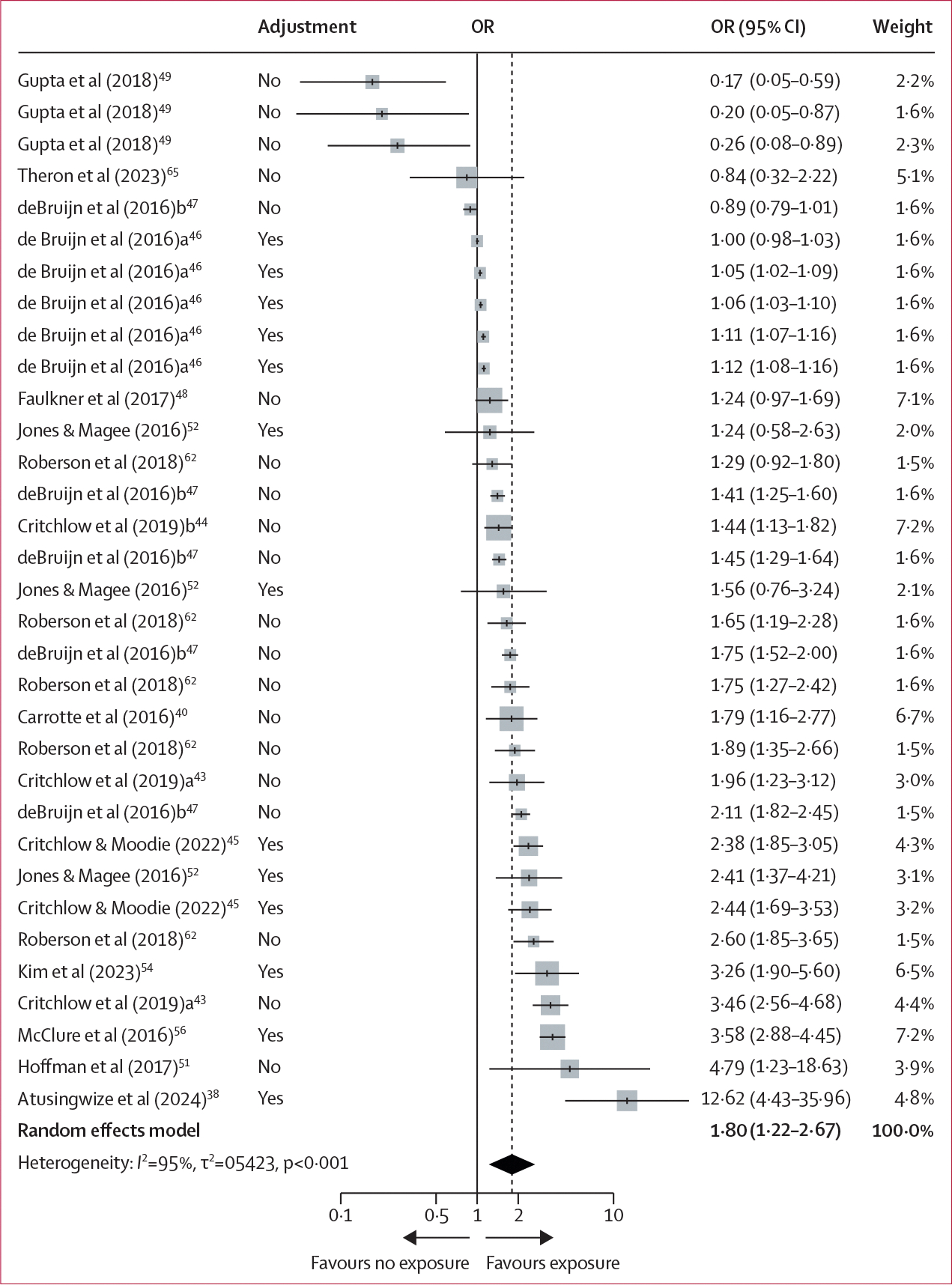
Forest plot of multilevel meta-analysis for exposure to digital alcohol marketing content and binge drinking Adjustment=whether or not a covariate adjustment was used to compute the OR. OR=odds ratio.

Participants who were exposed to digital alcohol marketing content, compared with those who were not exposed, had greater odds of reporting binge drinking (13 studies, 25 603 participants; OR 1·80 [95% CI 1·22–2·67]; *I*^2^=94·6%; figure 3). The estimated variance components were τ^2^_Level 3_=0·5003 and τ^2^_Level 2_=0·0421, corresponding to *I*^2^_Level 3_= 91·9% of the total variation attributed to between-cluster heterogeneity, and *I*^2^_Level 2_=7·7% to within-cluster heterogeneity. The three-level model provided a significantly better fit compared with a two-level model with level 3 heterogeneity constrained to zero (χ_1_^2^=20·03; p<0·01). Subgroup analyses showed that participants exposed to digital alcohol marketing content in the past 30 days, compared with those who were exposed in their lifetime, had greater odds of reporting binge drinking (appendix p 11).

**Figure 4:**
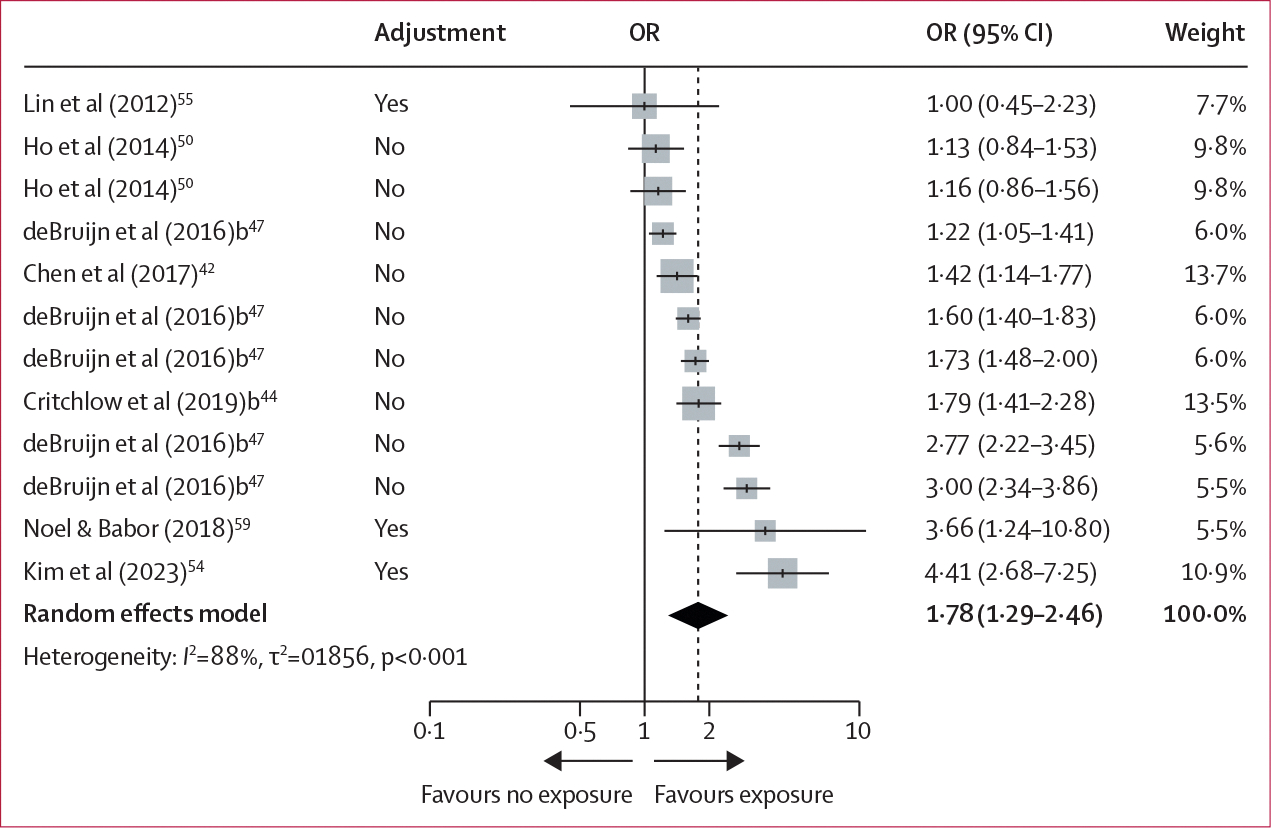
Forest plot of multilevel meta-analysis for exposure to digital alcohol marketing content and susceptibility to use alcohol among never users Adjustment=whether or not a covariate adjustment was used to compute the OR. OR=odds ratio.

Participants who were exposed to digital alcohol marketing content, compared with those who were not exposed, had greater odds of susceptibility to use alcohol among never users (seven studies, 18 698 participants; OR 1·78 [95% CI 1·29–2·46]; *I*^2^=88·0%; figure 4). The estimated variance components were τ^2^_Level 3_=0·0680 and τ^2^_Level 2_=0·1176, corresponding to *I*^2^_Level 3_=34·2% of the total variation attributed to between-cluster heterogeneity, and *I*^2^_Level 2_=59·1% to within-cluster heterogeneity. The three-level model did not provide a significantly better fit compared with a two-level model with level 3 heterogeneity constrained to zero (χ_1_^2^=0·26; p=0·61). However, the three-level model adequately represents the structure of the data and suggests that effects within studies were largely homogeneous. Due to a small number of effect sizes reported, subgroup analyses were not performed for the susceptibility to use alcohol measures among never users.

Participants who were exposed to digital alcohol marketing content, compared with those who were not exposed, had greater odds of reporting lifetime alcohol use (14 studies, 31 953 participants; OR 1·70 [95% CI 1·35–2·12]; *I*^2^=98·4%; appendix p 13). The estimated variance components were τ^2^_Level 3_=0·0883 and τ^2^_Level 2_=0·1554, corresponding to *I*^2^_Level 3_=36·0% of the total variation attributed to between-cluster heterogeneity, and *I*^2^_Level 2_=63·3% to within-cluster heterogeneity. The three-level model did not provide a significantly better fit compared with a two-level model with level 3 heterogeneity constrained to zero (χ_1_^2^=3·07; p=0·08). No significant differences between groups in the subgroup analyses of lifetime alcohol use were observed in this study (appendix p 12).

No statistically significant relationships were observed between design quality, sampling breadth, and alcohol use outcomes (appendix p 6). The Rücker limit meta-analysis method showed that bias adjusted (OR 1·71 [95% CI 1·52–1·93]; Z=8·80) and bias unadjusted (1·67 [1·53–1·86]; Z=10·36) random-effects models were similar. Evidential value was present (right-skewness test, p=0·001) in the p-curve analysis (appendix p 14), suggesting the presence of a true non-zero effect. Sensitivity analyses comparing adjusted and unadjusted models showed no statistically significant subgroup differences (appendix p 9). The risk of bias is visualised for each study in the appendix (p 15).

## Discussion

This study found that exposure to digital alcohol marketing content was associated with past 30-day alcohol use, binge drinking, susceptibility to use alcohol among never users, and lifetime alcohol use. Subgroup analyses indicated that adolescents (*vs* adults), individuals with past 30-day exposure (*vs* lifetime), and those exposed via social media platforms (*vs* other web-based platforms) had greater odds of reporting alcohol use.

Findings showed that adolescents exposed to digital alcohol marketing content, compared with adults, had greater odds of reporting past 30-day alcohol use. This finding adds quantitative evidence to previous qualitative reviews that have suggested a relationship between exposure to alcohol marketing and underage drinking.^5,7,9,69,70^ Digital platforms have increased reliance on artificial intelligence-driven algorithms to personalise and amplify content based on users’ past behaviours, interests, and engagement patterns.^13^ Moreover, social learning theory suggests that individuals learn behaviours by observing and modelling others, particularly when such behaviours appear to be rewarded or widely accepted.^71^ Social learning theory can help explain how digital marketing might shape adolescents’ and young adults’ intentions to drink by fostering observational learning and reinforcing pro-alcohol-related norms.^71,72^ Public health practitioners could consider this theory, and the unique features of social media marketing, when designing prevention programmes and interventions to counter the preponderance of digital alcohol marketing content online.

A large proportion of participants in this meta-analysis were adolescents (86·4%), a population for whom previous research has shown that exposure to advertising and alcohol portrayals in media increases the likelihood of later alcohol consumption.^6^ This heightened susceptibility among youth underscores the need for policy responses to restrict digital alcohol marketing to youth specifically. However, reliance on alcohol industry self-regulation has proven ineffective.^73,74^ To help address these gaps, WHO has developed guidance for comprehensive restrictions on digital alcohol marketing,^17,75^ including through its SAFER initiative, which calls for enforcing bans on alcohol marketing across digital platforms.

Building on these recommendations, a more robust youth-centred regulatory framework could include implementing independent age-verification systems, such as on alcohol brand websites and social media accounts, where pro-alcohol-related content is promoted. Existing age verification methods (eg, entering date of birth or checkboxes to confirm legal drinking age) are easily bypassed and often fail to prevent underage access.^16^ These methods could be improved by requiring users to upload a government-issued ID or by integrating third-party verification services to confirm age eligibility, particularly on alcohol brand websites and social media accounts, where underage users might encounter promotional content. International agreements could also be strengthened to enforce cross-border digital marketing restrictions. To hold industry accountable to standards outlined in the DISCUS guidelines (eg, requiring 73·8% of the audience to be of legal drinking age),^73^ enforceable penalties for non-compliance might be needed.

Our study has several limitations. The search was limited to peer-reviewed publications written in English, affecting its generalisability. Most of the studies included in this review relied on self-reported survey instruments, which could have increased the likelihood of self-report bias. For example, exposure to digital alcohol marketing was primarily measured through self-report, which could be affected by measurement biases, such as attentional and recall bias. Binge drinking was not measured to account for sex-based physiological differences, impacting its generalisability. Additionally, most included studies did not report results disaggregated by sex, gender, or ethnicity, limiting this study’s ability to examine potential subgroup differences or assess how structural and socioeconomic factors might have influenced the observed associations.

This meta-analysis included a combination of adjusted and unadjusted ORs, which might have introduced residual confounding. However, sensitivity analyses showed that adjusted and unadjusted estimates were highly consistent in magnitude and direction. Although this consistency across parameter estimates increased our confidence in the overall patterns of association, adjusted estimates remain preferable when available because they reduce potential confounding and strengthen the credibility of the findings. Only one study included in this review used an experimental design. As a result, this precluded analysing the causal impact of exposure to digital alcohol marketing content on alcohol use. 16 studies that met the inclusion criteria for this review were excluded from the final analysis due to non-responses from the corresponding authors, which might have impacted the parameter estimates.

Several studies in this meta-analysis included multiple platform-specific exposures in the same statistical model, which could have underestimated the unique effects of each platform (eg, Instagram *vs* TikTok) by forcing closely related variables to compete for shared variance. Because we relied on study-level data, we were unable to reconstruct composite measures for pooled analyses. Most studies included participants from adolescent or young adult samples which might not generalise to other age groups. This study was also limited by heterogeneity across included studies and variability in study quality. Differences in sample characteristics, exposure and outcome measures, and study design could have introduced inconsistencies that reduced the precision of pooled estimates.^76,77^ Additionally, the included studies varied in how they measured exposure (eg, dichotomous *vs* continuous scales) and often did not report specific levels of exposure to digital alcohol marketing content.

Exposure to digital alcohol marketing content is associated with alcohol use initiation and sustained alcohol use. Digital alcohol marketing content might normalise pro-alcohol-related behaviours among adolescents and young adults. Prevention programmes that promote abstention and strengthen digital media literacy might be critical to countering the effects of pro-alcohol content that exists online. Findings from this meta-analysis can inform the design of future prospective studies. Specifically, the effect size estimates can serve as a basis for power calculations and sample size estimation in longitudinal studies aimed at understanding similar associations. Future research should develop standardised measures or novel methodologies (eg, qualitative coding frameworks) of exposure to digital alcohol marketing. This could improve precision and reduce bias in exposure measurement, making findings comparable across studies. Future studies should also examine whether structural and individual factors, such as socioeconomic status, moderate the associations between digital alcohol marketing exposure and alcohol-related outcomes.

Our findings contribute to the growing body of evidence on the digital determinants of health, a domain recognising that digital environments, particularly those shaped by commercial actors, have a central role in shaping population health.^78^ Addressing digital alcohol marketing as a digital determinant of health is essential to designing effective policy responses that protect youth and promote equity.

## Supplementary Material

1

## Figures and Tables

**Table:** Study characteristics

	Design	Location	Population (N)	% Female	Digital platform	Exposure engagement	Exposure timeframe	Type of recall	Alcohol outcome	Effect size #

Atusingwize et al (2024)^38^	Cross-sectional	Uganda	996 young adults	45·9%	Social media	Active	Past 30 days	Yes/No	Past 30-day use; binge drinking	2
Bain et al (2023)^39^	Cross-sectional	Australia	3618 adolescents	58·9%	Web-based; social media	Passive	Past 30 days	Scale	Past 30-day use	2
Carrotte et al (2016)^40^	Cross-sectional	Australia	1001 adolescents and adults	72·2%	Social media	Active	Lifetime	Multiple response	Binge drinking; lifetime use	2
Chan et al (2024)^41^	Cross-sectional	China	675 adults	46·6%	Social media	Passive	Past 30 days	Yes/No	Past 30-day use	1
Chen et al (2017)^42^	Longitudinal	Taiwan	1863 adolescents	52·7%	Web-based	Passive	Past 30 days	Multiple response	Susceptibility to use; lifetime use	2
Critchlow et al (2019)a^43^	Cross-sectional	UK	3399 adolescents	49·0%	Social media	Active	Past 30 days	Multiple response	Past 30-day use; binge drinking	7
Critchlow et al (2019)b^44^	Cross-sectional	UK	3399 adolescents	49·0%	Social media	Passive	Past 30 days	Multiple response	Past 30-day use; binge drinking; susceptibility to use; lifetime use	4
Critchlow & Moodie (2022)^45^	Cross-sectional	Ireland	1007 adults	50·4%	Social media	Passive	Past 30 days	Scale	Past 30-day use; binge drinking	4
de Bruijn et al (2016)a^46^	Cross-sectional	Germany, Italy, Netherlands, and Poland	9038 adolescents	50·0%	Social media; web-based	Active; passive	Lifetime	Scale	Binge drinking; lifetime use	10
de Bruijn et al (2016)b^47^	Longitudinal	Germany, Italy, Netherlands, Poland	9075 adolescents	50·5%	Social media; web-based	Active; passive	Past 30 days	Yes/No	Past 30-day use; binge drinking; susceptibility to use; lifetime use	20
Faulkner et al (2017)^48^	Cross-sectional	Australia	4413 adolescents	51·7%	Web-based	Passive	Past 30 days	Multiple responses	Past 30-day use; binge drinking	2
Gupta et al (2018)^49^	Cross-sectional	Australia and India	631 (India, 330; Australia, 301) adolescents and young adults	India, 36·0%; Australia, 62·0%	Social	Active	Lifetime	Multiple responses	Past 30-day use; binge drinking; lifetime use	9
Ho et al (2014)^50^	Cross-sectional	Thailand	1028 adolescents	57·6%	Web-based	Active; passive	Past 30 days	Scale	Past 30-day use; susceptibility to use; lifetime use	6
Hoffman et al (2017)^51^	Cross-sectional	USA	637 adults	NA	Social media	Passive	Lifetime	Scale	Past 30-day use; binge drinking	2
Jones & Magee (2011)^52^	Cross-sectional	Australia	1113 adolescents	59·9%	Web-based	Passive	Lifetime	Multiple responses	Past 30-day use; lifetime use	2
Jones et al (2016)^53^	Cross-sectional	Australia	283 young adults	71·7%	Social media	Active	Lifetime	Multiple responses	Binge drinking; lifetime use	8
Kim et al (2023)^54^	Cross-sectional	China	675 young adults and adults	51·0%	Social media	Passive	Past 30 days	Yes/No	Past 30-day use; binge drinking; susceptibility to use	3
Lin et al (2012)^55^	Cross-sectional	New Zealand	2538 adolescents	49·0%	Social media; web-based	Active	Lifetime	Multiple responses	Susceptibility to use; lifetime use	6
McClure et al (2016)^56^	Longitudinal	USA	2012 adolescents	51·0%	Web-based	Passive	Lifetime	Yes/No	Binge drinking; lifetime use	2
McClure et al (2020)^57^	Cross-sectional	USA	202 adolescents	55·0%	Web-based	Passive	Lifetime	Yes/No	Lifetime use	1
McCreanor et al (2024)^58^	Cross-sectional	New Zealand	3698 adolescents	55·7%	Social media	Active; passive	Past 30 days	Yes/No	Lifetime use	2
Noel & Babor (2018)^59^	Experimental	USA	120 young adults	48·3%	Social media	Passive	Past 30 days	Scale	Susceptibility to use	1
Noel et al (2024)^60^	Cross-sectional	USA	824 young adults	44·9%	Social media: web-based	Passive	Past 30 days	Yes/No	Past 30-day use	3
Osuafor et al (2023)^61^	Cross-sectional	South Africa	3833 adolescents	52·2%	Social media	Passive	Lifetime	Yes/No	Lifetime use	1
Roberson et al (2018)^62^	Cross-sectional	USA	682 young adults	55·3%	Web-based; social media	Passive	Past 30 days	Scale	Binge drinking; lifetime use	10
Rutherford et al (2024)^63^	Cross-sectional	Australia	125 young adults	78·9%	Social media	Passive	Past 30 days	Yes/No	Past 30-day use	1
Steers et al (2024)^64^	Cross-sectional	USA	454 young adults	71·1%	Social media	Active	Past 30 days	Scale	Past 30-day use	3
Theron et al (2023)^65^	Cross-sectional	South Africa	792 adults	69·7%	Web-based	Passive	Lifetime	Scale	Binge drinking	1
Wang et al (2020)^66^	Cross-sectional	Mongolia	1277 young adults	23·7%	Web-based	Passive	Past 30 days	Yes/No	Past 30-day use; lifetime use	2
Yoshida et al (2023)^67^	Cross-sectional	Japan	15 683 adolescents	51·0%	Web-based	Active	Past 30 days	Yes/No	Past 30-day use	1
Zhang et al (2024)^68^	Longitudinal	China	49 young adults	67·0%	Social media: web-based	Passive	Past 30 days	Yes/No	Past 30-day use	2

Active=searching, posting, commenting, liking alcohol-related content. Adolescents=average age between 11 years and 17 years. Adults=sample included individuals aged above 25 years. Lifetime=exposure occurred more than a month ago. NA=not assessed. Passive=viewing advertisements, promotions, or alcohol-related coupons. Past 30 days=exposure occurred within the past 30 days. Web-based=internet, email, websites, smartphones. Young adults=average age between 18 years and 25 years.

* The amount of individual effect sizes that each study contributed.

## Data Availability

Data and associated study materials can be found at https://github.com/SDonaldsonRutgers/Alcohol_MetaAnalysis.

## Research in context

### Evidence before this study

We searched PubMed, PsychINFO, Web of Science, Scopus, Embase, and Communication & Mass Media for systematic reviews and meta-analyses examining the association between exposure to digital alcohol marketing and alcohol use, published from Jan 1, 2004 up to Feb 1, 2025. We limited our search to peer-reviewed English language publications and used the search terms: (digital alcohol marketing OR online alcohol advertising OR social media alcohol exposure) AND (alcohol use OR drinking behavior) AND (systematic review OR meta-analysis). Previous research has suggested that exposure to digital alcohol marketing was associated with increased alcohol consumption. However, previous studies have been either qualitative systematic reviews or observational studies focusing on individual populations. To our knowledge, no meta-analysis to date has quantitatively synthesised global evidence on this relationship or examined how methodological characteristics, including digital platform type, are associated with alcohol use behaviours.

### Added value of this study

To our knowledge, this study is the first to provide a quantitative synthesis of the association between digital alcohol marketing exposure and alcohol use outcomes. Using a multilevel random-effects meta-analysis of 31 studies comprising 62 703 participants across 17 countries, we found that exposure to digital alcohol marketing was associated with increased odds of past 30-day alcohol use, binge drinking, susceptibility to alcohol use among never users, and lifetime alcohol use.

### Implications of all the available evidence

The evidence highlights the need for experimental and longitudinal research that can clarify the temporal order around, and causal relationship between, exposure to digital alcohol marketing and alcohol-related behaviours.
