# Supplementary material for: Association between exposure to digital alcohol marketing and alcohol use: a systematic review and meta-analysis
Source: Lancet Public Health. Author manuscript; Available in PMC 2025 Dec 1. (PMC12667599; doi:10.1016/S2468-2667(25)00219-1)
Supplement: 1 [file NIHMS2119848-supplement-1.pdf]

THE LANCET
Public Health

Supplementary appendix

This appendix formed part of the original submission and has been peer reviewed. We post it as supplied by the authors.

Supplement to: Donaldson SI, Russell AM, La Capria K, et al. Association between exposure to digital alcohol marketing and alcohol use: a systematic review and meta-analysis. *Lancet Public Health* 2025; **10:** e912–22.

Supplementary Appendix

Supplementary Tables

Appendix A. PRISMA 2020 Checklist
Appendix B. Keyword search string for PubMed, PsychINFO, Web of Science, Scopus, Embase, Communication & Mass Media
Appendix C. Relationship between effect size, design quality, and sampling breadth
Appendix D. ROBINS-E risk of bias codebook
Appendix E. Subgroup analyses of covariate adjustment on effect size estimates
Appendix F. Subgroup analyses of past 30-day alcohol use
Appendix G. Subgroup analyses of binge drinking
Appendix H. Subgroup analyses of lifetime alcohol use

Supplementary Figures

Appendix I. Forest plot of multilevel meta-analysis for exposure to digital alcohol-related marketing content and lifetime alcohol use
Appendix J. P-curve analysis results
Appendix K. Risk of bias assessments using the ROBINS-E Tool

**Appendix A.** PRISMA 2020 Checklist			
**Section and Topic**	**Item #**	**Checklist item**	**Location where item is reported**
**TITLE**			
Title	1	Identify the report as a systematic review.	Page 1
**ABSTRACT**			
Abstract	2	See the PRISMA 2020 for Abstracts checklist.	Page 2
**INTRODUCTION**			
Rationale	3	Describe the rationale for the review in the context of existing knowledge.	Page 8
Objectives	4	Provide an explicit statement of the objective(s) or question(s) the review addresses.	Pages 8
**METHODS**			
Eligibility criteria	5	Specify the inclusion and exclusion criteria for the review and how studies were grouped for the syntheses.	Pages 8 and 9
Information sources	6	Specify all databases, registers, websites, organisations, reference lists and other sources searched or consulted to identify studies. Specify the date when each source was last searched or consulted.	Page 8
Search strategy	7	Present the full search strategies for all databases, registers and websites, including any filters and limits used.	Page 8
Selection process	8	Specify the methods used to decide whether a study met the inclusion criteria of the review, including how many reviewers screened each record and each report retrieved, whether they worked independently, and if applicable, details of automation tools used in the process.	Page 9
Data collection process	9	Specify the methods used to collect data from reports, including how many reviewers collected data from each report, whether they worked independently, any processes for obtaining or confirming data from study investigators, and if applicable, details of automation tools used in the process.	Page 9
Data items	10a	List and define all outcomes for which data were sought. Specify whether all results that were compatible with each outcome domain in each study were sought (e.g. for all measures, time points, analyses), and if not, the methods used to decide which results to collect.	Page 10
	10b	List and define all other variables for which data were sought (e.g. participant and intervention characteristics, funding sources). Describe any assumptions made about any missing or unclear information.	Page 13
Study risk of bias assessment	11	Specify the methods used to assess risk of bias in the included studies, including details of the tool(s) used, how many reviewers assessed each study and whether they worked independently, and if applicable, details of automation tools used in the process.	Pages 11 and 12
Effect measures	12	Specify for each outcome the effect measure(s) (e.g. risk ratio, mean difference) used in the synthesis or presentation of results.	Pages 11 and 12
Synthesis	13a	Describe the processes used to decide which studies were eligible	Pages

methods		for each synthesis (e.g. tabulating the study intervention characteristics and comparing against the planned groups for each synthesis (item #5)).	11 and 12
	13b	Describe any methods required to prepare the data for presentation or synthesis, such as handling of missing summary statistics, or data conversions.	Page 11 and 12
	13c	Describe any methods used to tabulate or visually display results of individual studies and syntheses.	Page 13
	13d	Describe any methods used to synthesize results and provide a rationale for the choice(s). If meta-analysis was performed, describe the model(s), method(s) to identify the presence and extent of statistical heterogeneity, and software package(s) used.	Page 13
	13e	Describe any methods used to explore possible causes of heterogeneity among study results (e.g. subgroup analysis, meta-regression).	Pages 12
	13f	Describe any sensitivity analyses conducted to assess robustness of the synthesized results.	Pages 12 and 13
Reporting bias assessment	14	Describe any methods used to assess risk of bias due to missing results in a synthesis (arising from reporting biases).	Pages 12 and 13
Certainty assessment	15	Describe any methods used to assess certainty (or confidence) in the body of evidence for an outcome.	NA
**RESULTS**			
Study selection	16a	Describe the results of the search and selection process, from the number of records identified in the search to the number of studies included in the review, ideally using a flow diagram.	Page 13
	16b	Cite studies that might appear to meet the inclusion criteria, but which were excluded, and explain why they were excluded.	Page 13
Study characteristics	17	Cite each included study and present its characteristics.	Page 13
Risk of bias in studies	18	Present assessments of risk of bias for each included study.	Pages 15 and 16
Results of individual studies	19	For all outcomes, present, for each study: (a) summary statistics for each group (where appropriate) and (b) an effect estimate and its precision (e.g. confidence/credible interval), ideally using structured tables or plots.	Pages 14 and 15
Results of syntheses	20a	For each synthesis, briefly summarise the characteristics and risk of bias among contributing studies.	Pages 15 and 16
	20b	Present results of all statistical syntheses conducted. If meta-analysis was done, present for each the summary estimate and its precision (e.g. confidence/credible interval) and measures of statistical heterogeneity. If comparing groups, describe the direction of the effect.	Pages 14 and 15
	20c	Present results of all investigations of possible causes of heterogeneity among study results.	Pages 14 and 15

	20d	Present results of all sensitivity analyses conducted to assess the robustness of the synthesized results.	Pages 14 and 15
Reporting biases	21	Present assessments of risk of bias due to missing results (arising from reporting biases) for each synthesis assessed.	Pages 15 and 16
Certainty of evidence	22	Present assessments of certainty (or confidence) in the body of evidence for each outcome assessed.	NA
**DISCUSSION**			
Discussion	23a	Provide a general interpretation of the results in the context of other evidence.	Page 16
	23b	Discuss any limitations of the evidence included in the review.	Pages 18, 19, and 20
	23c	Discuss any limitations of the review processes used.	NA
	23d	Discuss implications of the results for practice, policy, and future research.	Page 20
**OTHER INFORMATION**			
Registration and protocol	24a	Provide registration information for the review, including register name and registration number, or state that the review was not registered.	Page 8
	24b	Indicate where the review protocol can be accessed, or state that a protocol was not prepared.	Page 8
	24c	Describe and explain any amendments to information provided at registration or in the protocol.	NA
Support	25	Describe sources of financial or non-financial support for the review, and the role of the funders or sponsors in the review.	Page 13
Competing interests	26	Declare any competing interests of review authors.	Page 21
Availability of data, code and other materials	27	Report which of the following are publicly available and where they can be found: template data collection forms; data extracted from included studies; data used for all analyses; analytic code; any other materials used in the review.	Page 21

Appendix B. Keyword search string for PubMed, PsychINFO, Web of Science, Scopus, Embase, Communication & Mass Media

Concept	Search string for each concept	Complete search strategy
Digital media	Social media OR TikTok OR YouTube OR Instagram OR Facebook OR Snapchat OR Twitter OR WeChat OR WhatsApp OR Reddit OR Tumblr OR Flickr OR Pinterest OR Google Plus OR LinkedIn OR digital OR online OR internet OR email OR web* OR smartphone OR mass media	Social media OR TikTok OR YouTube OR Instagram OR Facebook OR Snapchat OR Twitter OR WeChat OR WhatsApp OR Reddit OR Tumblr OR Flickr OR Pinterest OR Google Plus OR LinkedIn OR digital OR online OR internet OR email OR web* OR smartphone OR mass media AND alcohol* OR liquor OR social drinking OR drinking behavior* OR binge drinking OR beer OR wine OR intoxicate* OR underage drink* AND marketing OR communication OR promotion OR advert* OR content OR brand OR sponsor Or product placement OR merchandis*
Alcohol	Alcohol* OR liquor OR social drinking OR drinking behavior* OR binge drinking OR beer OR wine OR intoxicate* OR underage drink*	
Marketing	Marketing OR communication OR promotion OR advert* OR content OR brand OR sponsor Or product placement OR merchandis*	

Appendix C. Relationship between effect size and design quality and sampling breadth

Variable	*k*	*OR*	95% *CI*	I^2, %	$p^{subgroup}$
Design quality					0.33
Cross-sectional/convenience	54	1.59	[1.36, 1.86]	83.7	
Cross-sectional/representative	35	1.86	[1.52, 2.27]	98.6	
Longitudinal/convenience	0	--	--	--	
Longitudinal/representative	32	1.75	[1.50, 2.05]	93.3	
Experimental/convenience	1	3.66	[1.24, 10.80]	--	
Sampling breadth					0.06
Local	40	1.68	[1.45, 1.96]	80.2	
State	14	2.31	[1.78, 3.00]	86.4	
National	68	1.63	[1.42, 1.87]	98.1	

Note. k = number of effect sizes; *OR* = odds ratio; *CI* = confidence interval; I^2 = the percentage of total variance attributable to heterogeneity between studies; $p^{subgroup}$ = test of subgroup differences.

**Appendix D.** ROBINS-E risk of bias codebook	
Domain	Definition
Bias Due to Confounding	Assesses whether key confounders (e.g., age, sex, socioeconomic status, sensation seeking, peer alcohol use) were appropriately measured and adjusted for in the analysis.<br>• Low risk: Study adjusted for key confounders (e.g., age, SES, peer alcohol use, sensation seeking) using multivariate modeling.<br>• Some concerns: Adjusted for some important confounders, but key ones are missing or not clearly defined.<br>• High risk: No confounding variables adjusted for; unadjusted ORs only or minimal covariate control.<br>• No information: Adjustment strategy not described or insufficient detail provided.
Bias Arising from Measurement of the Exposure	Assesses whether exposure to alcohol marketing was measured accurately and in a way that reflects true exposure, including the method and specificity of measurement.<br>• Low risk: Exposure measured using a validated instrument or digital trace data.<br>• Some concerns: Self-reported exposure using detailed, specific items (e.g., clearly defined timeframe and platform).<br>• High risk: Vague or general self-report of exposure (e.g., "Have you seen alcohol ads?"); likely recall or attention bias.<br>• No information: No clear description of exposure measurement.
Bias in Participant Selection	Assesses whether participants included in the analysis were representative of the population, and whether inclusion/exclusion criteria were clearly defined.<br>• Low risk: Nationally or regionally representative sample; random sampling procedures clearly described.<br>• Some concerns: Convenience sample with unclear representativeness; limited description of recruitment methods.<br>• High risk: Convenience sample, highly selective sample, or poor justification of inclusion/exclusion.<br>• No information: Sampling method and inclusion criteria not described.
Bias Due to Missing Data	Assesses whether the extent of missing data was reported and whether appropriate methods were used to address it.<br>• Low risk: Missing data reported and handled using appropriate statistical techniques (e.g., multiple imputation).<br>• Some concerns: Missing data reported but not addressed or handled using basic methods (e.g., complete case analysis).<br>• High risk: High levels of missing data or unclear handling; potential for bias.<br>• No information: No mention of missing data in the study.

Bias Arising from Measurement of the Outcome	Assesses whether the alcohol use outcome was measured using a valid and reliable approach, including clarity of definitions and time frames.<br>• Low risk: Outcome defined using standard timeframes and thresholds (e.g., past 30-day use, binge drinking defined by NIAAA cut-offs).<br>• Some concerns: Some ambiguity in definitions (e.g., “regular use,” “often drinks”), but sufficient to infer intent.<br>• High risk: Subjective or undefined outcome measures (e.g., “Do you drink alcohol?” with no timeframe).<br>• No information: Outcome measurement not described.
Bias in Selective Reporting	Assesses whether there was evidence of selective outcome reporting or omission of relevant outcomes from the analysis.<br>• Low risk: All outcomes described in the methods section are reported; protocol or pre-registration available.<br>• Some concerns: Minor discrepancies between stated and reported outcomes.<br>• High risk: Evidence of outcome switching, selective outcome omission, or unexplained exclusions.<br>• No information: Insufficient detail to evaluate selective reporting.

Appendix E. Subgroup analyses of covariate adjustment on effect size estimates

Variable	*k*	*OR*	95% *CI*	I^2, %	$p^{subgroup}$
Past 30 Days					0.58
Adjusted	13	1.74	[1.46, 2.06]	60.8	
Unadjusted	29	1.89	[1.47, 2.44]	93.4	
Binge Drinking					0.34
Adjusted	13	1.80	[1.29, 2.50]	95.4	
Unadjusted	20	1.48	[1.20, 1.84]	89.1	
Susceptibility to use					0.38
Adjusted	3	1.66	[1.32, 2.08]	79.3	
Unadjusted	9	2.56	[1.00, 6.56]	89.1	
Lifetime use					0.87
Adjusted	21	1.67	[1.28, 2.17]	98.9	
Unadjusted	14	1.62	[1.33, 1.97]	84.2	

Note. k = number of effect sizes; *OR* = odds ratio; *CI* = confidence interval; I^2 = the percentage of total variance attributable to heterogeneity between studies; $p^{subgroup}$ = test of subgroup differences; Adjusted = models that controlled for potential confounding variables such as age, sex, socioeconomic status, and other study-specific covariates; Unadjusted = models that reflected bivariate associations.

**Appendix F.** Subgroup analyses of past 30-day alcohol use					
Variable	k	*OR*	95% *CI*	I^2, %	$p^{subgroup}$
Engagement					0.10
Active	15	2.39	[1.56, 3.67]	93.1	
Passive	27	1.64	[1.41, 1.90]	87.7	
Exposure Timeframe					<0.01
Past 30 days	37	2.00	[1.66, 2.42]	91.5	
Lifetime	5	1.05	[0.77, 1.44]	61.0	
Platform					<0.01
Social media	26	2.21	[1.69, 2.90]	91.0	
Other web-based	16	1.43	[1.25, 1.64]	77.1	
Age					0.02
Adolescents	18	2.36	[1.74, 3.20]	94.7	
Adults	24	1.55	[1.27, 1.88]	79.9	

Note. k = number of effect sizes; *OR* = odds ratio; *CI* = confidence interval; I^2 = the percentage of total variance attributable to heterogeneity between studies; $p^{subgroup}$ = test of subgroup differences; Other web-based = internet, email, websites, smartphones; Active = searching, posting, commenting, liking digital alcohol-related content; Passive = viewing digital alcohol-reated content; Lifetime = exposure occurred more than a month ago; Past 30 days = exposure occurred within the past 30 days; adolescents = average age between 12 and 17; adults = average age above 18.

Appendix G. Subgroup analyses of binge drinking

Variable	k	*OR*	95% *CI*	I^2, %	$p^{subgroup}$
Engagement					0.49
Active	15	1.39	[0.89, 2.16]	94.0	
Passive	18	1.65	[1.35, 2.01]	95.0	
Exposure Timeframe					0.02
Past 30 days	18	1.87	[1.55, 2.25]	90.8	
Lifetime	15	1.15	[0.80, 1.65]	92.8	
Platform					0.47
Social media	20	1.67	[1.21, 2.29]	91.8	
Other web-based	13	1.44	[1.15, 1.81]	96.6	
Age					0.64
Adolescents	15	1.47	[1.18, 1.82]	96.6	
Adults	18	1.63	[1.12, 2.38]	76.3	

Note. k = number of effect sizes; *OR* = odds ratio; *CI* = confidence interval; I^2 = the percentage of total variance attributable to heterogeneity between studies; $p^{subgroup}$ = test of subgroup differences; Other web-based = internet, email, websites, smartphones; Active = searching, posting, commenting, liking digital alcohol-related content; Passive = viewing digital alcohol-related content; Lifetime = exposure occurred more than a month ago; Past 30 days = exposure occurred within the past 30 days; adolescents = average age between 12 and 17; adults = average age above 18.

**Appendix H.** Subgroup analyses of lifetime alcohol use					
Variable	k	*OR*	95% *CI*	I^2, %	$p^{subgroup}$
Engagement					0.27
Active	21	1.79	[1.39, 2.30]	93.6	
Passive	14	1.48	[1.16, 1.88]	99.1	
Exposure Timeframe					0.64
Past 30 days	12	1.74	[1.27, 2.38]	92.3	
Lifetime	23	1.59	[1.28, 1.98]	98.7	
Platform					0.23
Social media	16	1.87	[1.37, 2.57]	98.8	
Other web-based	19	1.50	[1.24, 1.82]	93.9	
Age					0.58
Adolescents	25	1.62	[1.29, 2.01]	98.9	
Adults	10	1.91	[1.46, 2.09]	0.0	

Note. k = number of effect sizes; *OR* = odds ratio; *CI* = confidence interval; I^2 = the percentage of total variance attributable to heterogeneity between studies; $p^{subgroup}$ = test of subgroup differences; Other web-based = internet, email, websites, smartphones; Active = searching, posting, commenting, liking digital alcohol-related content; Passive = viewing digital alcohol-related content; Lifetime = exposure occurred more than a month ago; Past 30 days = exposure occurred within the past 30 days; adolescents = average age between 12 and 17; adults = average age above 18.

Supplementary Figures

Appendix I. Forest plot of multilevel meta-analysis for exposure to digital alcohol-related marketing content and lifetime alcohol use.

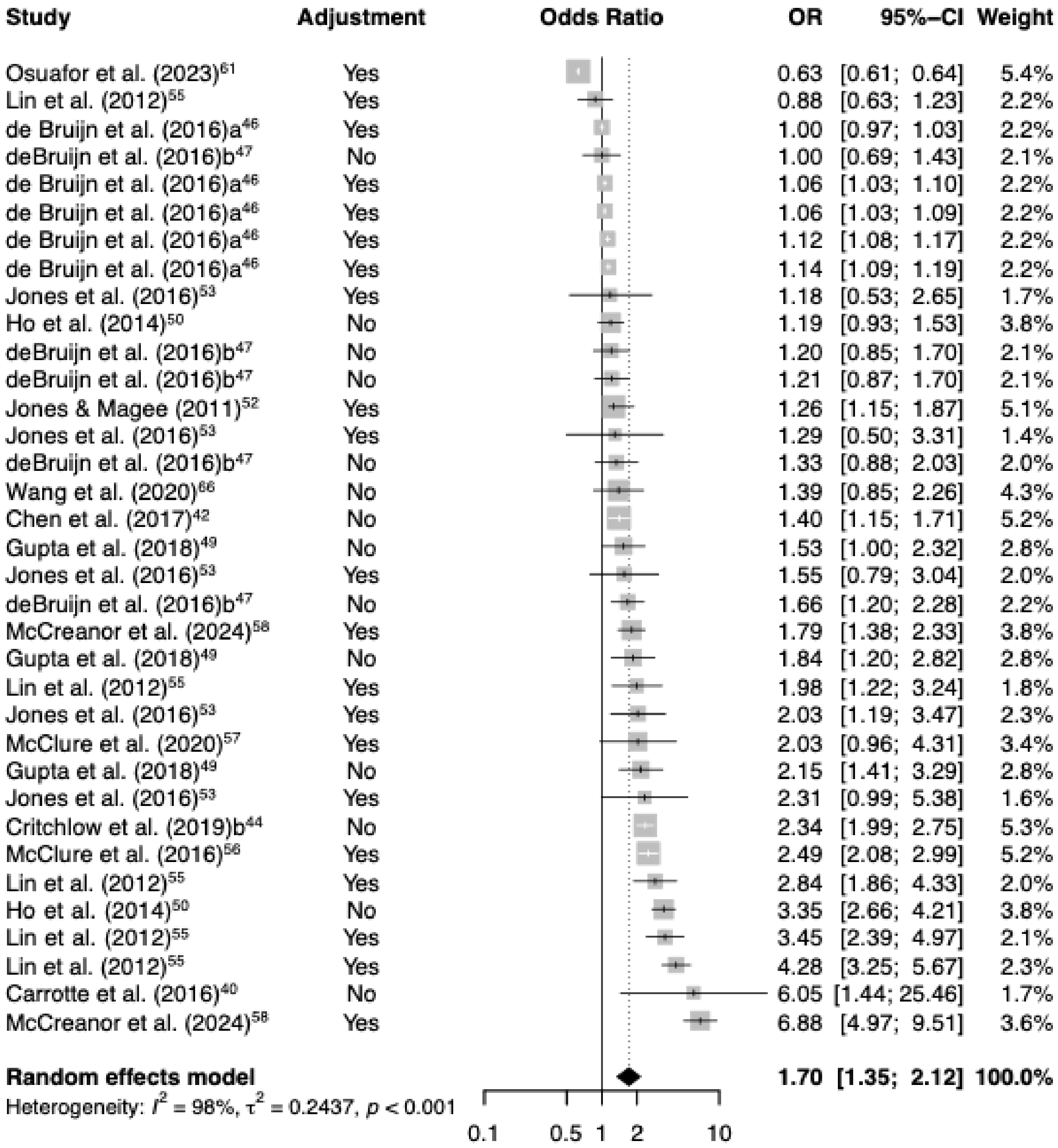

Note. Adjustment = whether or not a covariate adjustment was used to compute the odds ratio; OR = odds ratio; CI = confidence interval.

Appendix J. P-curve analysis results.

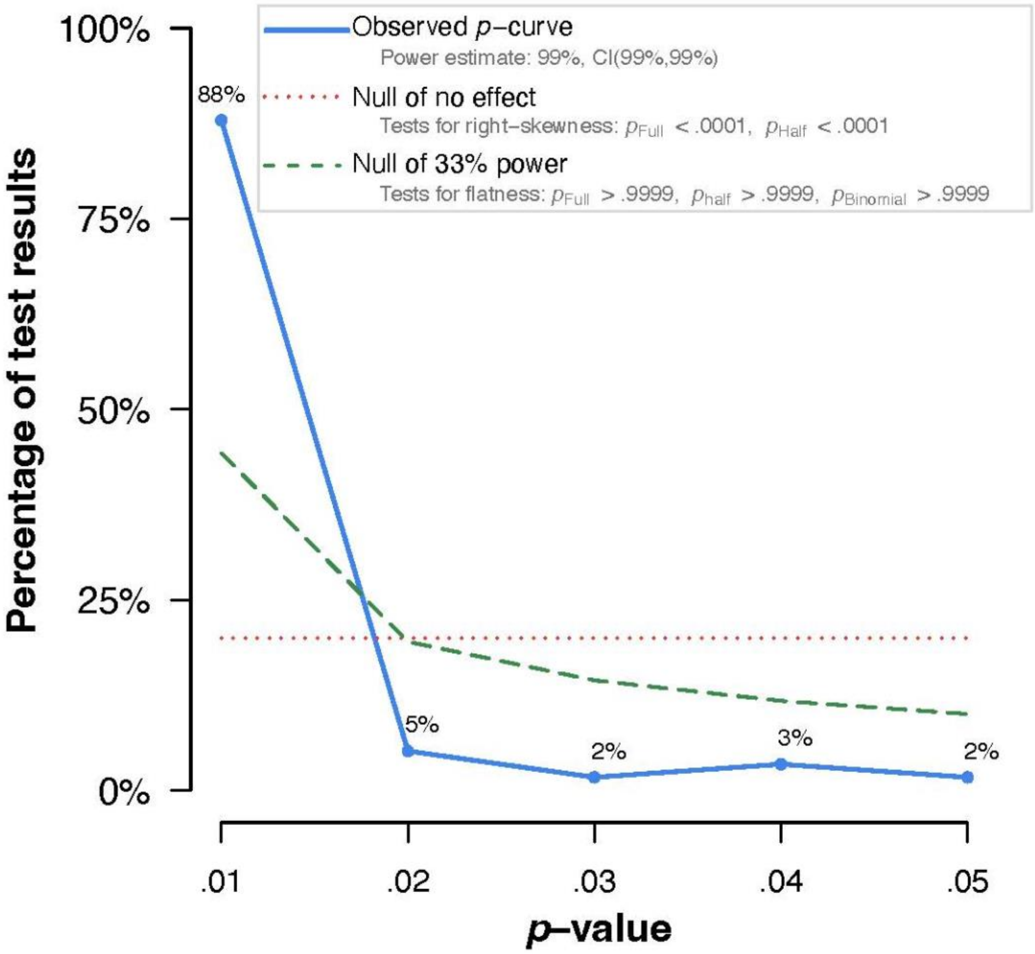

Note: The observed p−curve includes 58 statistically significant ($p < .05$) results, of which 54 are p
< There were 27 additional results entered but excluded from p−curve because they were $p > .05$.

Appendix K. Risk of bias assessments using the ROBINS-E Tool

Risk of bias domains

Study	D1	D2	D3	D4	D5	D6	D7	Overall
Atusingwize et al. (2025)	Low	Some concerns	Some concerns	Not applicable	Low	No information	Low	Some concerns
Carrotte et al. (2016)	High	High	High	Not applicable	Low	No information	Low	High
Chan et al. (2024)	High	Low	Some concerns	Not applicable	Some concerns	No information	Low	High
Chen et al. (2017)	High	Low	Some concerns	Not applicable	Low	Low	Low	High
Critchlow & Moodie (2022)	Low	Low	Low	Not applicable	Low	Low	Low	Low
Critchlow et al. (2019)a	Low	Low	Low	Not applicable	Low	Low	Low	Low
Critchlow et al. (2019)b	Low	Low	Low	Not applicable	Low	Low	Low	Low
de Bruijn et al. (2016)a	Low	Low	Low	Not applicable	Low	No information	Low	No information
deBruijn et al. (2016)b	Low	Low	Low	Not applicable	Low	Low	Low	Low
Faulkner et al. (2017)	Some concerns	Low	Low	Not applicable	Low	No information	Low	No information
Gupta et al. (2018)	High	High	Some concerns	Not applicable	Low	Low	Low	High
Ho et al. (2014)	High	Some concerns	Low	Not applicable	Some concerns	Low	Low	High
Hoffman et al. (2017)	High	High	Low	Not applicable	Low	No information	Low	High
Jones & Magee (2011)	Low	Some concerns	High	Not applicable	Low	No information	Low	High
Jones et al. (2016)	Low	High	Some concerns	Not applicable	Low	Low	Low	High
Kim & Chan (2023)	Low	Low	Low	Not applicable	Low	No information	Low	No information
Lin et al. (2012)	Low	Low	Low	Not applicable	Some concerns	No information	Low	No information
McClure et al. (2016)	Low	Low	Low	Not applicable	Low	Low	Low	Low
McClure et al. (2020)	Low	Some concerns	Low	Not applicable	Low	Low	Low	Some concerns
McCreanor et al. (2024)	Low	Some concerns	Low	Not applicable	Low	Some concerns	Low	Some concerns
Noel & Babor (2018)	Low	High	Low	Not applicable	Some concerns	Low	Low	High
Noel et al. (2024)	Low	Low	Some concerns	Not applicable	Some concerns	No information	Low	No information
Osuafor et al. (2023)	Low	Low	High	Not applicable	Some concerns	No information	Low	No information
Roberson et al. (2018)	High	Some concerns	Low	Not applicable	Low	No information	Low	No information
Rutherford et al. (2024)	High	Some concerns	Low	Not applicable	Low	No information	Low	No information
Steers et al. (2024)	High	Some concerns	Low	Not applicable	Low	Low	Low	High
Theron et al. (2023)	High	Some concerns	Some concerns	Not applicable	Low	No information	Low	High
Wang et al. (2020)	High	Some concerns	Some concerns	Not applicable	Low	No information	Low	High
Yoshida et al. (2023)	Low	Low	Some concerns	Not applicable	Low	Low	Low	Some concerns
Zhang et al. (2024)	Low	Some concerns	Low	Not applicable	Low	No information	Low	No information

Domains:
D1: Bias due to confounding.
D2: Bias arising from measurement of the exposure.
D3: Bias in selection of participants into the study (or into the analysis).
D4: Bias due to post−exposure interventions.
D5: Bias due to missing data.
D6: Bias arising from measurement of the outcome.
D7: Bias in selection of the reported result.

Judgement
- High
- Some concerns
- Low
- No information
- Not applicable

Note. The fourth domain (Bias due to departures from intended exposures) was deemed not applicable across studies because most of the included studies were observational.
